# The Identification and Differentiation between *Burkholderia mallei* and *Burkholderia pseudomallei* Using One Gene Pyrosequencing

**DOI:** 10.1155/2014/109583

**Published:** 2014-10-02

**Authors:** Damian H. Gilling, Vicki Ann Luna, Cori Pflugradt

**Affiliations:** Center for Biological Defense, College of Public Health, University of South Florida, Tampa, FL 33612, USA

## Abstract

The etiologic agents for melioidosis and glanders,* Burkholderia mallei* and* Burkholderia pseudomallei* respectively, are genetically similar making identification and differentiation from other* Burkholderia* species and each other challenging. We used pyrosequencing to determine the presence or absence of an insertion sequence* IS*407A within the flagellin P (*fli*P) gene and to exploit the difference in orientation of this gene in the two species. Oligonucleotide primers were designed to selectively target the* IS*407A-*fli*P interface in* B. mallei* and the* fli*P gene specifically at the insertion point in* B. pseudomallei*. We then examined DNA from ten* B. mallei*, ten* B. pseudomallei,* 14* B. cepacia*, eight other* Burkholderia* spp., and 17 other bacteria. Resultant pyrograms encompassed the target sequence that contained either the* fli*P gene with the* IS*407A interruption or the fully intact* fli*P gene with 100% sensitivity and 100% specificity. These pyrosequencing assays based upon a single gene enable investigators to reliably identify the two species. The information obtained by these assays provides more knowledge of the genomic reduction that created the new species* B. mallei *from* B. pseudomallei* and may point to new targets that can be exploited in the future.

## 1. Introduction

The proteobacteria* Burkholderia mallei* and* Burkholderia pseudomallei*, distinct from the* Burkholderia cepacia* complex, cause melioidosis and glanders, respectively [1]. Inhalation of either organism can lead to pneumonia with reported mortality rates of 19–50% [2, 3]. Although human-to-human spread is extremely rare,* B. mallei* is highly infectious when aerosolized and resulting infections can be debilitating, painful, and difficult to diagnose in humans [4]. Historically,* B. mallei* was used as a bioweapon during the Civil War and the two World Wars with suspected use by the former USSR in Afghanistan [4–6]. Infections with* B. pseudomallei *are more common especially in endemic areas such as Southeast Asia and Northern Australia [7]. In the United States, rare infections with* B. pseudomallei* have been reported; usually patients are military personnel who were exposed when they were on active duty in endemic areas either recently or even as long as 62 years before [8, 9]. Two active disease cases were reported in US civilians who had traveled to Honduras where they presumably were exposed [10] (CDC, 2006). Other cases have been reported in Arizona, Puerto Rico, and the British Virgin Islands as recently as 2011 where the source of infection appeared to be local soils and flood waters [11–15]. Laboratory exposure has also been reported in California and Florida [10, 16]. Today, both* B. mallei* and* B. pseudomallei* are classified as Tier 1 select agents by the Centers for Disease Control and Prevention (CDC) due to their high virulence via the respiratory route, as well as potential to cause illness on a large scale if ever widely disseminated [6].

Symptoms of* B. pseudomallei* infection vary, ranging from severe pneumonia with concomitant abscesses in the liver and spleen to persistent infections of the skin, soft tissues, bones, and joints [2]. Often the symptomology of* B. pseudomallei *infections parallel to those of infections such as tuberculosis which makes empiric diagnosis difficult unless the organism can be isolated, identified, and confirmed in the laboratory [2, 17, 18]. Another factor which makes the clinical identification and differentiation between* B. pseudomallei* and* B. mallei* even more challenging is the fact that human cases of glanders and melioidosis infections are not common in nonendemic parts of the world. As a result many medical professionals in clinics and laboratories lack experience with these bacteria [18, 19]. Additionally,* B. pseudomallei* has earned the nickname “the great mimicker” due to its wide repertoire of clinical manifestations which often prompt misdiagnosis. Several rapid detection/diagnostic biochemical methods have been introduced; yet problems have been reported such as the misidentification of* B. pseudomallei* as* B. cepacia* [19–23]. Thus research has shifted focus toward molecular methods, but these too have had their problems, primarily assay specificity and sensitivities which are less than 100% [24, 25]. Culture methods have traditionally been the gold standard for identifying* B. mallei* and* B. pseudomallei* [26, 27]. However these methods can be time consuming. Many molecular methods focus on* B. mallei* and the loss of a gene or phenotype and appear to be reliable in identifying* B. mallei* but none of these methods can consistently distinguish* B. pseudomallei* from other* Burkholderia* species [19, 25]. DNA fingerprinting assays such as multilocus sequence typing (MLST) have been proven efficacious at detection but are also time consuming, expensive, and technically challenging for many laboratories [28]. Identification of* B. mallei* and* B. pseudomallei* through the sequencing of 16S DNA targets has been successfully accomplished; however, discrimination of the two closely related species requires additional sequencing [29, 30].

Genetically,* B. pseudomallei* and* B. mallei* are so similar that differentiating between the two is a challenge. In fact* B. mallei* evolved directly from* B. pseudomallei* due to a process called reductive evolution wherein a massive invasion of many insertion sequence (*IS*) elements altered the* B. pseudomallei* genome through genomic rearrangements and deletions. This reduction evolution resulted in a smaller genome with enough key genotypic and phenotypic differences to create a separate species now known as* B. mallei* [31, 32]. The numerous* IS* elements in the* B. mallei *genome account for about 3.1% of the total genome; yet this smaller genome is only 80% in size of that of* B. pseudomallei* [32, 33]. One specific insertion sequence, known as* IS*407A can be located in multiple sites in the genome and has been found to interrupt the flagellar gene (*fli*P) that encodes the flagellar P protein and renders* B. mallei* nonmotile [33]. Additionally, the resultant truncated* fli*P gene is completely flipped, having undergone a complete inversion on the DNA strand. This genomic structure and nonmotility is found in all* B. mallei* [32]. In comparison,* B. pseudomallei* does not contain the* IS*407A insertion sequence within its* fli*P gene. This complete gene confers full motility and contains sequence regions that are distinct from the* fli*P gene in other* Burkholderia species* such as* B. cepacia.*

The* B. mallei fli*P/*IS*407A region with its species wide conserved gene inversion and truncation provided the target for* B. mallei* used in this work. We hypothesized that when the target region was sequenced, that sequence would exhibit both the end of the IS407A insertion and a section of the* fli*P gene that would be in the reverse orientation to that same gene found in* B. pseudomallei*. We then chose a* B. pseudomallei* target that covered the same section of the* fli*P gene where the* IS*407A insertion sequence was located in* B. mallei*, using the rationale that, when sequenced, this target will exhibit either (A) a complete* fli*P match in the correct orientation and thus can be identified as* B. pseudomallei *or (B) nothing at all if the gene is interrupted which precluded the primers from binding. Thus this scheme is able to detect and differentiate between* B. pseudomallei* and* B. mallei*. We hypothesized that the targeting of the* fli*P gene with its clear differences between* B. mallei* and* B. pseudomallei* and distinctions from other* Burkholderia* spp. should provide a reliable form of confirmation between inconclusive results.

The purpose of this study was to explore the use of pyrosequencing to exploit the genetic difference of the* fli*P gene, its orientation, and surrounding sequences in* B. mallei* and* B. pseudomallei* as a single gene target method of detection and differentiation between the two species and other* Burkholderia* species. We wanted to determine if the pyrosequencing platform, that is, putatively simple, quick, and trustworthy [34], would enable the consistent and reliable microbial typing by targeting conserved* fli*P regions of* B. pseudomallei* in addition to the variable* fli*P/*IS*407A regions of* B. mallei*.

## 2. Materials and Methods

### 2.1. Bacterial Strains

We examined ten* Burkholderia mallei*, ten* Burkholderia pseudomallei*, and 40 other bacterial strains, including 15* B. cepacia*, 3* B. thailandensis,* and five various* Burkholderia* species (Table 1). The* B. mallei*,* B. pseudomallei,* and 2* B. thailandensis* were received from the NIH Biodefense and Emerging Infections Research Resources Repository (BEI Resources, Bethesda, MD, USA) as either live strains or purified DNA. Access to both isolates and DNA of both* B. mallei* and* B. pseudomallei *were limited due to restrictions on how many Tier 1 isolates and DNA can be purchased yearly from BEI Resources. Of the 15* B. cepacia* isolates, one was obtained from the American Type Culture Collection (ATCC, Manassas, VA, USA), three were collected from the Florida Department of Health, Bureau of Laboratories, Tampa, FL (FDOH) and 11 were procured from the University of Washington Medical Center in Seattle, WA. The other 23 bacterial strains including six various* Burkholderia* species were part of our large bacterial collection and were either collected previously as clinical isolates from FDOH or received from ATCC (Table 1).

All manipulations of cultures of* B. mallei *and* B. pseudomallei *strains were performed in a biological safety cabinet in a biological safety level 3 (BSL3) laboratory. All safety protocols followed “Biosafety in Microbiological and Biomedical Laboratories, 5th Edition” (BMBL) practices including the use of protective laboratory clothing and respiratory equipment [35]. The safety and security requirements by US federal regulation DHHS 42 CFR 73 were strictly adhered to. Manipulations of the other bacterial strains were performed in a biological safety level 2 (BSL2) laboratory in a biological safety cabinet following safety practices outlined in BMBL as above.

### 2.2. DNA Extraction

Bacteria were grown on tryptic soy agar supplemented with 5% sheep red blood cells (blood agar (BA)) (Remel, Lenexa, KS, USA). All culture plates were incubated at 35°C overnight (18–24 hours) before performing DNA extractions. Following manufacturers' instructions, all genomic DNA extractions were either performed using the Epicentre (Qiagen Inc, Madison, WI) extraction kits, a MagNaPure Compact automated instrument (Roche, Inc, Indianapolis, IN, USA), or a boil preparation method used by FDOH. The boil preparation, (adapted from a reference lab in the Laboratory Response Network) is described as follows: bacterial growth from an overnight cultured media plate was removed and placed into 100 *μ*L of sterile water and boiled for 5 minutes and then placed onto ice for 2 minutes, followed by centrifugation at 12,000 ×g for 10 minutes at 4°C. The supernatant was transferred to a 0.1 *μ*M filter tube (Millipore Corporation Billerica, MA) and centrifuged for 2 minutes at 8,000 ×g. To prove sterility of all filtrates and DNA extractions (following the University of South Florida Institutional Biosafety Committee guidelines), 10 *μ*L was sacrificed and used to inoculate a BA media culture plate and incubated at 35°C for 48 hours. Extracts and lysates having no growth after two days were allowed out of the BSL3 environment and made available for molecular work. The DNA was stored at 4°C or −30°C until used. Before use, the DNA was diluted 1 : 20 in molecular-grade water (Fisher Scientific, Pittsburg, PA) to achieve a working concentration of 0.5 to 2 ng/*μ*L.

### 2.3. Validation PCR Assays and Gel Electrophoresis

**Figure 1:**
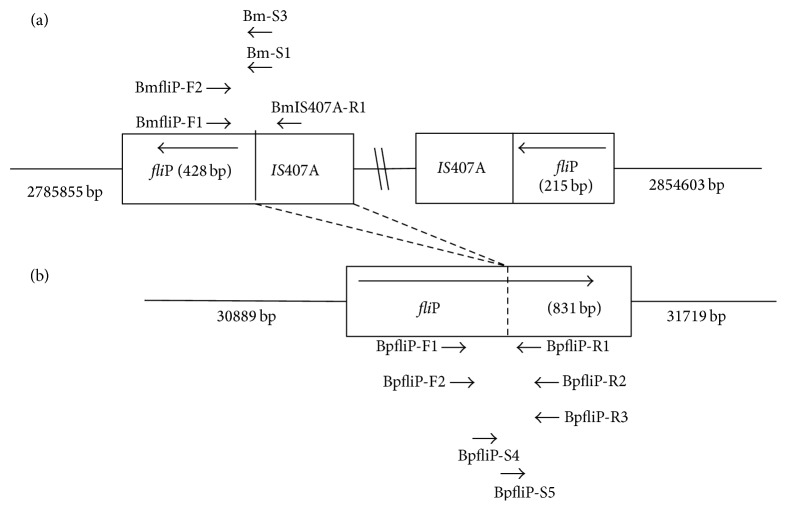
(a) Schematic of* Burkholderia mallei fli*P gene with* IS*407A interruption that caused both gene inversion and loss of function. The large arrow within the block designates the orientation of the two parts of the* fli*P gene on the DNA strand. The location and direction of the various primers are noted by the small arrows. The pyrosequencing target in* B. mallei* is the interface between the insertion sequence element and* fli*P gene. (b) Schematic of the* Burkholderia pseudomallei* intact and functional* fli*P gene. The large arrow within the block designates the orientation of the intact gene while the location and directions of the primers used are denoted by the small arrows. The pyrosequencing target in* B. pseudomallei* covers both sides of the point of the* fli*P gene that corresponds to where the insertion sequence is found in* B. mallei*.

Based upon the* fli*P gene GenBank sequence BMA2686, PCR assays were designed to target sequences of the* fli*P gene in* B. mallei *(NCBI GenBank Accession #:NC_006348) and* B*.* pseudomallei* (NCBI GenBank Accession #:NC_009076).  Figure 1 shows a schematic of the target regions in both species. All oligonucleotides used in the PCR assays for* B. mallei *and* B pseudomallei* are listed in Table 2. Primer-BLAST analysis (via NCBI) predicted 217 bp and 397 bp amplicons for the* fli*P—*IS*407A interface in* B. mallei *and the* fli*P gene in* B psudomallei,* respectively. The PCR reaction mixture with a final volume of 10 *μ*L volume consisted of 1 *μ*L of DNA template (at a concentration of 0.5–2 ng/*μ*L), 1 *μ*L of the Takara (Takara, Madison, WI, USA) 10X buffer containing 1.5 mM MgCl_2_, 0.82 *μ*L of dNTPs at 25 *μ*M, 0.05 *μ*L of Takara HS Taq enzyme, and 2 *μ*L of each specific primer at a concentration of 1 *μ*M. Universal primers for the 16S rDNA sequence of* Burkholderia* spp. previously described [20, 30] were later incorporated in the assays and produced a 1500 bp product to act as a control. The universal primers were IAC-16S-F (5′-AGAGTTTGATCCTGGCTCAG-3′) and IAC-rDNA-R (5′-ACGGCTACCTTGTTACGACTT-3′). All PCR reactions were carried out on a T1 Biometra Thermocycler (Biometra Inc., Göttingen, Germany) with the following conditions: initial denaturation at 95°C for 15 minutes, followed by 35 cycles of 94°C for 30 seconds, 58°C for 1 minute, and 72°C for 3 minutes, and lastly a final extension of 72°C for 10 minutes.

PCR amplicons were electrophoresed on a 1% agarose gel containing 15 *μ*g of ethidium bromide (final concentration of 0.2 *μ*g/mL) in 1X TBE (44.5 mmol Tris-borate and 1 mmol EDTA, pH 8.3) for 60 min at 100 mV constant voltage. DNA bands were visualized with UV light and photographed using the GelDoc (Bio-Rad, Hercules, CA, USA).

### 2.4. Sanger Sequencing

The amplicons from the validation PCR and the initial pyrosequencing PCR reactions were purified using the Wizard DNA Purification Kit (Promega, Madison, WI, USA). Cycle sequencing was performed using the Beckman-Coulter Dye Terminator Cycle Sequencing (DTCS) Quick Start kit protocol (Beckman-Coulter, Fullerton, CA, USA). Cycle sequencing reactions with a final volume of 20 *μ*L consisted of 8 *μ*L DTCS Quick Start master mix (Beckman-Coulter), 1.5 *μ*L sequence primer at 25 pmol, and 8 *μ*L of DNA. Cycle sequencing PCR was performed with the Biometra T3 Thermocycler (Biometra Inc.,) for 30 cycles under the following conditions: 96°C for 20 seconds, 50°C for 20 seconds, and 60°C for 4 minutes. The DNA produced from the cycle sequencing reaction was precipitated as per the Beckman-Coulter ethanol precipitation protocol. Finally, Sanger sequencing was performed on a Beckman-Coulter CEQ-8000 (Fullerton, CA) following the manufacturer's directions. The resulting nucleotide sequences were compared to the public databases via NCBI nucleotide nBLAST alignment which confirmed or negated the sequences' identity for either* B. mallei* or* B. pseudomallei*.

### 2.5. Pyrosequencing

Initial pyrosequencing PCR assays with the validation primers described above for* B. mallei* and* B. pseudomallei *were carried out in 50 *μ*L reaction volumes. The amplicons were then used as template for the pyrosequencing assays using primers that nested within the amplicon sequence (Table 2 and Figure 1). One primer from each primer set carried a biotin tag at the 5′ end (Table 2). Different primer sets were used to examine if the target sequence could be produced consistently or if some primer locations were better than others. Finally, sepharose beads were used to capture the remaining biotinylated PCR product, after which the product-bead mixture was washed using a series of solutions that removed impurities and stripped the double-stranded DNA down to single-stranded DNA. This was done using the Pyromark vacuum prep worktable (Qiagen, Valencia, CA, USA). Specific pyrosequencing primers (Table 2) designed to target the regions of interest on* B. mallei* and* B. pseudomallei* were then annealed to the single-stranded DNA template before analysis on the Q96 ID Pyrosequencer (Qiagen). This procedure was performed as per the manufacturer's instructions with each sample sequenced at least twice. The assay was conducted in the sequence analysis (SQA) mode and the pyrosequencing results were graded as pass or fail based on the test samples' adherence to the predetermined qualifications as per the manufacturer's directions. All strains were tested a minimum of two times to confirm results.

## 3. Results and Discussion

In the validation PCR assays, the* fli*P target regions of* B. mallei* and* B. pseudomallei* were successfully amplified. The* fli*P target gene region for* B. mallei* produced a 217 bp PCR product as predicted while the assay for the* B. pseudomallei* target region produced a 397 bp amplicon (data not shown). All ten* B. mallei* amplicons from the PCR assays when sequenced using the Sanger sequencing method had 100% nucleotide identity with the target sequence (*B. mallei fli*P gene and insertion sequence in GenBank accession NC_006348). When sequenced using the Sanger sequencing method, all ten amplicons produced from the* B. pseudomallei* DNA templates displayed 100% nucleotide identity with the published target gene (*B. pseudomallei fli*P gene sequence GenBank accession NC_009076).* B. cepacia* which possesses its own version of the* fli*P gene per published GenBank sequences have little to no nucleotide identity for either* B. mallei* or* B. pseudomallei* primers. As predicted none of the* B. cepacia* DNA templates nor the DNA from the six various* Burkholderia* spp. produced amplicons when used in either of the two PCR assays. Additionally, the DNA templates from the remaining bacterial strains, including the seven bacterial collection strains which possess their own versions of the* fli*P genes, yielded negative results.

When the DNA from the ten* B. mallei* strains were pyrosequenced with* B. mallei* pyrosequencing primers (Table 2) all ten tests were noted as “PASSED” and the resulting pyrograms were confirmed via NCBI nucleotide nBLAST alignment (Table 3). The NCBI nBLAST alignments confirmed that the pyrosequencing sequences matched with 100% nucleotide identity to publicly available* fli*P sequences of* B. mallei*. Different combinations of primer sequences (Table 2) were utilized to explore a larger target sequence and also resulted in 100% nucleotide identity no matter if the target was shifted slightly up or down stream (Figure 1). When the DNA from the ten* B. mallei* strains were pyrosequenced with the* B. pseudomallei* pyrosequencing primers (Table 2), all of the pyrograms were noted as “FAILED.” When the DNA from the ten* B. pseudomallei* strains were pyrosequenced using the* B. pseudomallei* sequencing primers (Table 2), all ten were noted as “PASSED” and when tested with the* B. mallei* pyrosequencing primers they were noted as “FAILED.” The resulting “PASSED” pyrograms were confirmed via NCBI nucleotide nBLAST and again matched with 100% nucleotide identity to published* B. pseudomallei fli*P sequences. The various primer sets that were designed and used (Table 2) generated sequences that shifted slightly up and down stream (Figure 1). The average pyrosequencing result obtained for the* B. pseudomallei* DNA templates was 44 nucleotides long (like the* B. mallei* results) and all had 100% nucleotide identity to* B. pseudomallei* GenBank sequences of the* fli*P gene and not for* fli*P gene sequences of other* Burkholderia* species. Repeated testing gave the same results. As displayed in Table 3 the pyrosequencing results for* B. mallei* and* B. pseudomallei* show the highest percentage matches when applied to an NCBI BLAST search. Both assays demonstrated 100% sensitivity as they reliably and consistently identified the known* B. mallei* or* B pseudomallei* DNA. This pyrosequencing method also demonstrated 100% specificity against the DNA samples of 15* B. cepacia* and six other* Burkholderia* spp. All yielded negative PCR results and failed pyrosequencing readouts with both of the* B. mallei* and* B. pseudomallei* pyrosequencing primer sets (Table 4). All pyrosequencing nucleotide sequences will be submitted for entry into the NCBI databases.

Reliable detection and differentiation of* B. mallei* and* B. pseudomallei* continue to be challenging because of the difficulty in developing a consistently sensitive, selective, and accurate assay [25]. Current molecular techniques such as ribotyping, and multilocus sequence typing are employed for detection and differentiation, but these methods can be labor intensive and challenging for laboratories and their usefulness is questionable in distinguishing differences between* B. mallei* and* B. pseudomallei* [19, 28]. Here we present a new method for detection and differentiation based on single gene target pyrosequencing. Pyrosequencing is alternative sequencing method, which provides real-time read-out, that is, highly suitable for sequencing short stretches of DNA [36]. Other key advantages of pyrosequencing include simple frequency data and the ability to generate sequence signals immediately downstream of the primer. Additionally, both sample preparation and single-strand DNA processing are relatively rapid [37]. Furthermore, pyrosequencing eliminates the need for labeled primers, dNTPs, or gel electrophoresis. We chose pyrosequencing as our target platform because it requires the least amount of sample manipulation, while still providing real-time read-out results that are highly suitable for sequencing short stretches of DNA (≤40 nucleotides). However, the target sequences and the assays described in this paper can be adapted to standard and real-time PCR in those laboratories that do not possess a pyrosequencer. In our experience pyrosequencing is cost-effective in testing samples when compared to qPCR, standard PCR, and dideoxynucleotide chain-terminating sequencing methods. Cost analysis was not in the scope of our paper but could be addressed by other researchers.

Clinically, when infection is suspected and before any treatment options can be explored proper pathogen identification is essential. This ensures that both the proper treatment and safety protocols are followed to protect both patient and healthcare practitioner. We proposed an effective method of detection and positive identification of* B. mallei* and* B. pseudomallei* based upon the overall genomic plasticity and multiple* IS* elements separating* B. mallei *from* B. pseudomallei* [32, 38]. The* IS*407A insertion that causes the* fli*P gene truncation, inversion, and subsequent loss of flagella functionality in* B. mallei *provided the target for reliable identification of both species. Our pyrosequencing assays targeted a short nucleotide sequence that encompassed both the inverted* fli*P sequence as well as the* IS*407A sequence. As this sequence is unique to* B. mallei* it was our assertion that this sequence should provide 100% identification of* B. mallei.* Examination of DNA from ten unique* B. mallei* isolates validated this claim. The fully functional, nontruncated, noninverted* fli*P gene of* B. pseudomallei *also provided a reliable target for identification. We focused on the region of the gene where the* IS*407A insertion sequence apparently was introduced to create the* B. mallei fli*P/*IS*407A interface.

The* Burkholderia* species contain several genes including* fli*P that contribute to the biosynthesis and function of the flagellar organelle [39]. While the* fli*P gene is present throughout the various* Burkholderia* species, minor sequence differences enabled us to employ specific targeting.* B. thailandensis *is genetically and phenotypically the closest neighbor to both* B. mallei* and* B. pseudomallei *[40]. However,* B. thailandensis* is avirulent towards humans and animals and therefore only three were examined in this study.* B. cepacia,* (a common opportunistic pathogen) is also genetically related to* B. mallei* and* B. pseudomallei *as well as familiar to clinicians and laboratorians in the United States. In addition,* B. cepacia* carries a version of the* fli*P gene and has been mistaken for* B. pseudomallei *[19]. Thus we added more* B. cepacia *isolates to our examination of the* B. pseudomallei *assay.* B. cepacia* does not contain any* IS* element in its* fli*P gene and thus we predicted that none of the DNA from these isolates would generate positive results with the* B. mallei *primers. The results validated our prediction. The* B. cepacia* DNA also produced negative results in the assays with the* B. pseudomallei* primers even though the* B. cepacia fli*P gene is fully functional. Alignment comparison between* B. pseudomallei* (NCBI GenBank Accession #:NC_009076) and* B. cepacia fli*P gene resulted in up to 88% alignment matches (NCBI sequence alignment). Disruption of the* fli*P gene gives* B. mallei* its distinct nonmotile phenotype, while the differences between* B. cepacia* and* B. pseudomallei fliP* genes provide genotypic targeting while still retaining their motility phenotype. While the* B. cepacia fli*P is fully functional, the sensitivity of pyrosequencing enables the exploitation of even a 12% difference because the oligonucleotides used in our pyrosequencing assay had low homology with the* B. cepacia fli*P sequence. It is only through sequencing that one can definitively differentiate between* B. pseudomallei *and* B cepac*ia. Our detection assays can enable the laboratory to identify an isolate as either* B. mallei or B. pseudomallei* with the confidence of eliminating false positives with* B. cepacia*. If positive for either select agent, the laboratory can immediately send the isolate to a Laboratory Reference Network (LRN) laboratory where it can be further examined and the CDC contacted if necessary. Although* B. cepacia* is not a focus of LRN laboratories, it is still a serious concern for the clinical laboratory and clinicians. Therefore a new pyrosequencing assay to reliably and consistently identify* B. cepacia *is being explored by our laboratory.

To date, we have not yet identified in the literature any* B. mallei* strains that do not have the* IS*407A element disrupting the* fli*P gene. Historically, this interruption with the subsequent rearrangement of the gene partial sequences' orientation and loss of genetic material has been one feature that distinguishes the* B. mallei *species from* B. pseudomallei.* Additionally, the element seems to be stable in* B. mallei* strains although it can theoretically move. Researchers have suggested that* B. mallei *evolved from a sole strain of* B. pseudomallei *and that the genome is “closed” and in “an evolutionary bottleneck in the mammalian host” that offered no opportunity for new genes [41]. It would be very interesting to see if in the future any* B. mallei* isolates have lost the* IS* element adjacent to the partial* fli*P gene sequence. If and when that occurs new assays can be designed using the partial* fli*P sequence and whatever DNA sequence remains after the exit of the* IS* element.

With new detection assays there is always a concern that since many are developed using pure culture they are susceptible to variable results when challenged with mixed cultures. Clinical samples are routinely cultured in nutrient rich media to identify pathogens and by custom and necessity to isolate each potential pathogen. Our study is designed for usage in clinical applications after the organism has been isolated from a clinical sample such as blood, skin, or deep tissue wound and the organism is not yet identified. So the fact that our assay was developed using pure cultures should have no negative impact on future applications. However, in the future, we plan to examine the possibility of direct detection straight from a clinical sample without using culturing and to test mixtures of bacteria, samples, and specimens such as swamp and irrigation waters, wounds, and sputum to explore if our assays can be utilized without the necessity of culturing to isolate the potential pathogen.

Although PCR based assays are prized for their high sensitivity this can also be a potential drawback. One of the primary drawbacks of PCR assay is “carry-over” products resulting from subsequent PCR runs which then become contaminants producing false positives [42, 43]. Pyrosequencing requires no initial PCR amplification or subsequent post PCR manipulation. The target sample DNA can be utilized directly as the template. Here we performed PCR only as an experimental validation step in the beginning of the study and later switched to extracted genomic DNA as template. The ability of pyrosequencing to identify short nucleotide sequences gives our assays specificity vital to the successful identification and differentiation of the two* Burkholderia* species. It is important to note that this assay is a starting point which will require further validation with the necessary cost analysis. In particular as both* B. mallei* and* B. pseudomallei* are Tier 1 designees access to multiple strains of DNA is strictly limited. Thus we could not test more isolates as would be required by the CDC. However, we are confident that this work is a starting point that focuses on a single gene as a target and can be useful to other researchers whether in the clinical or basic research laboratory.

## Figures and Tables

**Table 1:** List of bacteria used in this study.

Organism	CBD number^a^	Historical strain identifier
*Burkholderia *		
*Burkholderia mallei *	BB 0044	China 5 (MM-A, NBL 4) ATCC 10399
*Burkholderia mallei *	BB 0045	Ivan (NCTC 10230) ATCC 15310
*Burkholderia mallei *	BB 0046	China 7 (NBL 7) ATCC 23344
*Burkholderia mallei *	BB 0050	GB8 horse 4
*Burkholderia mallei *	DD-675	BURK014
*Burkholderia mallei *	DD-671	BURK005 (SR092700A)
*Burkholderia mallei *	DD-372	BURK007 (SR092700C)
*Burkholderia mallei *	DD-673	BURK009 (SR092700E)
*Burkholderia mallei *	DD-677	BURK062 (Turkey 1)
*Burkholderia mallei *	DD-178	BURK010 (2344)
*Burkholderia pseudomallei *	BB 0047	China 3 (MP-H, NBL 104)
*Burkholderia pseudomallei *	BB 0048	S 397 (NRRL B-1112, CCEB 472)
*Burkholderia pseudomallei *	BB 0049	286 (MP-S, NBL 121)
*Burkholderia pseudomallei *	BB 0051	K96243
*Burkholderia pseudomallei *	BB 0052	1026b
*Burkholderia pseudomallei *	BB 0053	1106b
*Burkholderia pseudomallei *	BB 0054	1710a
*Burkholderia pseudomallei *	BURK088	BURK088
*Burkholderia pseudomallei *	BURK099	BURK099
*Burkholderia pseudomallei *	NR-9320	K96423
*Burkholderia cepacia *	CBD 1341	FL-M-05-0506072
*Burkholderia cepacia *	CBD 1342	FL-M-05-B24210
*Burkholderia cepacia *	CBD 1343	FL-M-05-B25995R
*Burkholderia cepacia *	CBD 1442	ATCC BAA-245
*Burkholderia cepacia *	CBD 1450	F68492
*Burkholderia cepacia *	CBD 1456	H27659
*Burkholderia cepacia *	CBD 1455	H35975
*Burkholderia cepacia *	CBD 1449	M25311
*Burkholderia cepacia *	CBD 1458	M27066
*Burkholderia cepacia *	CBD 1451	M42544
*Burkholderia cepacia *	CBD 1453	M52455
*Burkholderia cepacia *	CBD 1454	M74393
*Burkholderia cepacia *	CBD 1457	T10400
*Burkholderia cepacia *	CBD 1452	T33589
*Burkholderia cepacia *	CBD 1448	T47491
*Burkholderia graminis *	CBD 1440	ATCC 700544
*Burkholderia multivorans *	CBD 1443	ATCC BAA-247
*Burkholderia stabilis *	CBD 1441	ATCC BAA-67
*Burkholderia vietnamiensis *	CBD 1438	ATCC 55792
*Burkholderia vietnamiensis *	CBD 1444	ATCC BAA-248
*Burkholderia thailandensis *	CBD 1439	ATCC 700388
*Burkholderia thailandensis *	BURK254	E254
*Burkholderia thailandensis *	BURK43	MSMB043
Other bacteria		
*Achromobacter xylosoxidans *	CBD 0307	ATCC 27061
*Acinetobacter baumannii *	CBD 1323^b^	ATCC 19606
*Acinetobacter calcoaceticus *	CBD 1336^b^	ATCC 23055
*Bacillus badius *	CBD 0091	ATCC 6462
*Cedecea neteri *	CBD 0309	ATCC 33855
*Citrobacter freundii *	CBD 0553^b^	ATCC 8090
*Enterobacter cloacae *	CBD 0556^b^	ATCC 13047
*Enterococcus faecalis *	CBD 1406	ATCC 49532
*Enterococcus faecalis *	CBD 1407	ATCC 49533
*Klebsiella pneumoniae *	CBD 0555^b^	ATCC 35657
*Lactobacillus rhanmosus *	CBD 1409	ATCC 53103
*Pseudomonas aeruginosa *	CBD 0551^b^	ATCC 15442
*Serratia marcescens *	CBD 1239	ATCC 13880
*Staphylococcus aureus *	CBD 0534	WA-HMC-03-4905
*Staphylococcus aureus *	CBD 1276	ATCC BAA-976
*Stenotrophomonas maltophilia *	CBD 0552^b^	ATCC 51331
*Streptococcus pneumoniae *	CBD 1405	ATCC 700669

^a^CBD: CBD stands for “Center for Biological Defense” and is the starting designation we use for bacterial strains that are in our BSL2 bacterial collection. Besides the CBD letter designation, each strain in our collection is also given a number to identify it. Each isolate in our BSL3 bacterial collection has a BB letter designation and then assigned a number as the BSL2 strains.

^b^denotes an isolate carries a homolog of the *fli*P gene.

**Table 2:** List of oligonucleotide primers used in pyrosequencing assays.

Organism	Target	Primer	Sequence (5′-3′)	Location (bp)^a^
*B. mallei *	*fli*P	BmfliP-F1^b^	ACGAACAGCGTGAGGAAGAG	2786291–2786310
	*IS*407A	BmIS407A-R1	CTAGAAGCCCATTGGCCCTAT	2786443–2786423
	*Interface *	Bm-S1	GGGGCAGGTCAACGA	2786417–2786403
	*Interface *	Bm-S3	GGCAGGTCAACGAGC	2786415–2786401

*B. mallei *	*fli*P	BmfliP-F2^b^	CGAACAGCGTGAGGAAGAG	2786292–2786310
	*IS*407A	BmIS407A-R1	CTAGAAGCCCATTGGCCCTAT	2786443–2786423
	*Interface *	Bm-S3	GGCAGGTCAACGAGC	2786415–2786401

*B. pseudomallei *	*fli*P	BpfliP-F1	AGACGATGCTGCTGCTCAC	31112–31130
	*fli*P	BpfliP-R1^b^	CCCGACGAGCACCTGATTC	31257–31239
	*fli*P	BpfliP-R2^b^	GAACAGCGTGAGGAAGAGGG	31281–31262
	*fli*P	BpfliP-S4	GCTGTCGTTCCTGCC	31134–31148

*B. pseudomallei *	*fli*P	BpfliP-F2^b^	GACGATGCTGCTGCTCAC	31113–31130
	*fli*P	BpfliP-R3	AACAGCGTGAGGAAGAGGG	31280–31262
	*fli*P	BpfliP-S5	AGCAGCGACAGCACG	31205–31191

Pyrosequencing primer sets for *B. mallei* and *B. pseudomallei* with respective target regions. Primers were designed using the design software supplied by the manufacturer.

^a^denotes location on the published sequences of either *B. mallei* GenBank accession number NC_006348 or *B. pseudomallei* GenBank accession number NC_009076.

^b^denotes a biotinylated primer.

“Interface” denotes the sequence where *IS*407A interrupts the *fli*P gene.

“F” denotes forward primer used for PCR.

“R” denotes reverse primer used for PCR.

“S” denotes the pyrosequencing primers.

The primers were first rehydrated in molecular grade water to bring them to a 100 *μ*M concentration for each primer. Forward and reverse primers were used at 1 *μ*M concentration while the sequence primers were used at 100 *μ*M.

The target sequence for the primer sets for *B. mallei* was: GCCTGCCGCAGCAGCGACAGCACGACGATGATCCGCGTGA, located at 2786360–2786399 bp in the sequence NCBI Genbank Accession #: NC_006348. The target sequence for the primer sets for *B. pseudomallei* was: GCGATGCTGCTGATGATGACGAGCTTCACGCGGATCATCA, located in the NCBI Genbank Accession #: NC_009076 at 31150–31189 bp. PCR primer targets for both *B. mallei* and *B. pseudomallei* are all located on their respective chromosome 1. All primer coordinates were last verified on April 1, 2014.

**Table 3:** Results of NCBI BLASTs of Sequences obtained by Pyrosequencing Assay.

Organism name	CBD Number	NCBI GenBank accession number
*Burkholderia mallei *	BB 0044	CP000548.1 *Burkholderia mallei *
*Burkholderia mallei *	BB 0045	CP000548.1 *Burkholderia mallei *
*Burkholderia mallei *	BB 0046	CP000548.1 *Burkholderia mallei *
*Burkholderia mallei *	BB 0050	CP000548.1 *Burkholderia mallei *
*Burkholderia mallei *	DD-675	CP000548.1 *Burkholderia mallei *
*Burkholderia mallei *	DD-671	CP000548.1 *Burkholderia mallei *
*Burkholderia mallei *	DD-372	CP000548.1 *Burkholderia mallei *
*Burkholderia mallei *	DD-673	CP000548.1 *Burkholderia mallei *
*Burkholderia mallei *	DD-677	CP000548.1 *Burkholderia mallei *
*Burkholderia mallei *	DD-178	CP000548.1 *Burkholderia mallei *
*Burkholderia pseudomallei *	BB 0047	CP006470.1 *Burkholderia pseudomallei *
*Burkholderia pseudomallei *	BB 0048	CP006470.1 *Burkholderia pseudomallei *
*Burkholderia pseudomallei *	BB 0049	CP003781.1 *Burkholderia pseudomallei *
*Burkholderia pseudomallei *	BB 0051	CP006470.1 *Burkholderia pseudomallei *
*Burkholderia pseudomallei *	BB 0052	CP003781.1 *Burkholderia pseudomallei *
*Burkholderia pseudomallei *	BB 0053	CP006470.1 *Burkholderia pseudomallei *
*Burkholderia pseudomallei *	BB 0054	CP006470.1 *Burkholderia pseudomallei *
*Burkholderia pseudomallei *	BURK088	CP006470.1 *Burkholderia pseudomallei *
*Burkholderia pseudomallei *	BURK099	CP006470.1 *Burkholderia pseudomallei *
*Burkholderia pseudomallei *	NR-9320	CP006470.1 *Burkholderia pseudomallei *

Outcome of pyrosequencing results compared against the NCBI database for alignment matches for either *B. mallei* or *B. pseudomallei*. Only the first match for each pyrosequence output is listed in the table. No sequences were obtained in any tests with *B. cepacia* or other bacterial strains and therefore no blast-searches were performed.

**Table 4:** Results of PCR and pyrosequencing assays for *B. mallei* and *B. pseudomallei. *

Organism name (number)	*B. mallei* Assays		*B. pseudomallei* Assays	
	PCR	Pyrosequence	PCR	Pyrosequence
*B. mallei* (10)	+	**PASS**	−	FAIL
*B. pseudomallei* (10)	−	FAIL	+	**PASS**
*B. cepacia* (15)	−	FAIL	−	FAIL
*Burkholderia* species (8)	−	FAIL	−	FAIL
Other Bacteria (17)				
Gram negative strains (10)	−	FAIL	−	FAIL
Gram positive strains (7)	−	FAIL	−	FAIL

“+” denotes a positive result or amplicon was produced.

“−” denotes a negative results.

“PASS” denotes the pyrosequencing assay produced a reliable sequence.

“Fail” denotes the pyrosequencing assay produced no reliable sequence or no sequence at all.
